# Varying Cognitive Scars – Differential Associations Between Types of Childhood Maltreatment and Facial Emotion Processing

**DOI:** 10.3389/fpsyg.2020.00732

**Published:** 2020-04-16

**Authors:** Benjamin Iffland, Frank Neuner

**Affiliations:** Department of Psychology, Bielefeld University, Bielefeld, Germany

**Keywords:** child maltreatment, attentional bias, face in the crowd effect, emotion recognition, visual search

## Abstract

**Background:**

Distorted cognitive processing has been found among survivors of child maltreatment. However, different types of abuse and neglect may bring about differences in emotion and attention processing. The present study aimed to detect differential associations between various types of childhood maltreatment and attentional biases in facial emotion processing.

**Methods:**

A non-clinical sample was recruited on University campus and consisted of 67 individuals with varying degrees of maltreatment. In an evaluative conditioning task, images of faces with neutral emotional expressions were either associated with short videos of intense negative statements, or associated with neutral videos. Subsequently, these faces were used as stimuli in a face in the crowd recognition task in which the familiar faces had to be recognized within a crowd of unfamiliar neutral faces.

**Results:**

In multiple linear regression analyses controlling for the intercorrelatedness of types of maltreatment, differential relationships between types of maltreatment and attentional bias were found. While emotional abuse was associated with faster detection of negatively associated faces, emotional neglect was associated with an impaired recognition of familiar stimuli regardless of the emotional content.

**Conclusion:**

Results indicated that interindividual differences in cognitive biases may be due to the activation of diverse cognitive schemas based on differential experiences of maltreatment.

## Introduction

Attentional biases are characterized by a selectively and differentially allocated attention toward emotional stimuli in comparison to neutral stimuli (for a review, see Cisler and Koster, 2010). In addition to the fact that attentional biases are a robust phenomenon among clinical populations (e.g., Bar-Haim et al., 2007; Cisler and Koster, 2010), recent studies have indicated that experiences of childhood adversities are also associated with distorted attentional processes (e.g., Günther et al., 2015). Previous studies consistently showed attentional biases in the processing of threatening information among individuals who reported adverse child experiences. For example, healthy individuals with a history of childhood maltreatment were more sensitive in detecting threatening cues from emotionally ambiguous faces, suggesting facilitated processing of threatening information (Pollak and Sinha, 2002; Gibb et al., 2009). Additionally, altered attentional processing of negative emotional cues as indicated by attentional avoidance of threatening faces and difficulties in disengaging from sad faces has been reported in samples of children who had been physically abused (Pine et al., 2005; Romens and Pollak, 2012).

In clinical samples, attentional biases were found in patients with posttraumatic stress disorder who reported sexual abuse in their childhood and adolescence but not in patients who did not report sexual abuse (Field et al., 2001). This finding indicates that experiences of child maltreatment may outweigh the effects of psychiatric diagnoses as correlates of attentional biases. Consistent with this assumption, attentional avoidance of emotional stimuli was more closely linked to the experience of emotional maltreatment than to the current diagnostic status in a sample of psychiatric inpatients and healthy controls (Iffland et al., 2019).

However, child maltreatment is a heterogenous phenomenon that includes different types of abuse and neglect. A growing body of literature indicated that various forms of childhood maltreatment have differential effects on psychopathology including depression and personality disorders (Danielson et al., 2005; Teicher et al., 2006; Lobbestael et al., 2010; Teicher and Samson, 2013).

In line with this, differentiated effects of abusive vs. neglectful environments on neural mechanisms linking childhood maltreatment to psychopathology and difficulties in emotional functioning have been reported recently (Dong et al., 2004; McLaughlin et al., 2014; Sheridan and McLaughlin, 2014; Humphreys and Zeanah, 2015; Zeanah and Sonuga-Barke, 2016; Roth et al., 2018). Hence, it is likely to assume that attentional biases toward threatening stimuli may be influenced differently by different forms of maltreatment. Nonetheless, little is known about the relationship beween different types of maltreatment and neurocognitive processes. So far, the current literature is limited by either incorporating only physical (Pollak and Sinha, 2002; Pine et al., 2005; Grant et al., 2011; Romens and Pollak, 2012), emotional (van Harmelen et al., 2013; Iffland et al., 2019), or sexual (Field et al., 2001) types of abuse, or by summing up the different types of childhood maltreatment without further differentiation (Gibb et al., 2009). Only a few studies have examined differential effects. Günther et al. (2015) analyzed the impact of five factors of childhood maltreatment (emotional abuse, emotional neglect, physical abuse, physical neglect, and sexual abuse) on attentional biases separately. In their dot-probe task, maintained attention toward sad faces was associated with emotional forms of maltreatment and physical neglect, but not with physical and sexual abuse (Günther et al., 2015). Most notably, although they examined a sample of depressed individuals, the association of maltreatment and sustained attention was not confounded by the severity of symptoms of depression. Likewise, varied biases and errors in the processing of emotional cues as a function of type of maltreatment could be shown in a facial emotion recognition task (Pollak et al., 2000). Here, physically neglected children showed difficulties in differentiating among emotional expression, while physically abused children revealed a response bias for angry facial expressions. However, contradictory results were presented by Fani et al. (2011). Although childhood maltreatment uniquely predicted attentional biases toward happy faces relative to neutral faces in a sample of PTSD patients, the authors could not detect differing associations between face processing and different types of childhood abuse. Hence, the extent to which the various types of childhood maltreatment account for differences in attentional biases still remains an open question. Moreover, given that previous studies examined adults suffering from clincial depression (Günther et al., 2015), or PTSD (Fani et al., 2011), or maltreated children (Pollak et al., 2000), the purpose of the present study was to extend the previous research by examining differential associations between various types of childhood maltreatment and emotion processing biases independent of psychopathology in a non-clinical adult sample.

As an experimental task of attentional biases we applied a visual search task (for a review see Cisler and Koster, 2010) that requires participants to detect familiar stimuli within a context of distraction stimuli. Previous research with a variant of this paradigm, the face in the crowd (FIC) experiment, has consistently documented that angry faces are detected more efficiently than happy faces among neutal control faces (Hansen and Hansen, 1988; Öhman et al., 2001; Horstmann and Bauland, 2006). This effect was referred to as the “anger superiority effect” or “face in the crowd effect” (FICE; Pinkham et al., 2010). From an evolutionary perspective it has been proposed that this effect reflects a fitness advantage for the quick location, recognition, and response to potential environmental threats (Öhman et al., 2001; Horstmann and Bauland, 2006). In a further elaboration of this evolutionary theory it may be assumed that the processes underlying the FICE are sensitive to environmental experiences. Hence, the repetetive confrontation with a stimuli indicating threat during development as it occurs in child maltreatment may shape the attention system to prioritize these stressors. Accordingly, the FICE was suggested to be related to amygdala activity which in turn has been shown to be hyper-responsive in maltreated individuals (Öhman and Mineka, 2001; Grant et al., 2011; Dannlowski et al., 2013; van Harmelen et al., 2013). Moreover, maltreated individuals showed increased sensitivity and vigilance toward emotional stimuli (Heim et al., 2000; Kendall-Tackett, 2000; Obradović et al., 2012; Voellmin et al., 2015).

Most studies utilizing visual search paradigms in facial emotion processing used schematic faces as stimuli (e.g., Öhman et al., 2001; Fox et al., 2010) which allows to control perceptual differences between emotional expressions. However, studies utilizing more realistic images of real faces showed inconclusive results regarding the FICE (Gilboa-Schechtman et al., 1999; Juth et al., 2005; Williams et al., 2005; Fox and Damjanovic, 2006; Horstmann and Bauland, 2006). It has also been argued that the use of pictures of real faces as stimuli does not necessarily increase external validity (Pinkham et al., 2010) since the presentation of multiple pictures of single identities results in unnatural homogeneous crowds. To overcome this limitation, Pinkham et al. (2010) used face images of multiple identities to create a more heterogeneous and realistic crowd consisting of both male and female faces. In addition, they allowed for any combination of emotional expressions of targets and distractors to avoid priming effects. The findings supported the FICE also in an experimental design with an enhanced ecological validity (Pinkham et al., 2010).

In a further extension of the evolutionary theory of attentional biases, we postulate that not only the detection of emotional expressions, but also the detection of identities associated with different emotions may be shaped by life experiences. In real-world settings threat is not always associated with overt facial expressions of negative emotions as anger or disapproval. Depending on previous experiences with specific persons, the rapid identification of potential perpetrators surrounding us rather than perceptual features of their faces should initiate a quick location, recognition, and response to potential social threats. This may be even more relevant for subjects with a history of childhood maltreatment, particularly emotional maltreatment. It may be assumed that emotional types of maltreatment, such as verbal hostility, taunting, belittling, rejection (Egeland, 2009), increase the sensitivity of victims’ response to social cues evaluated as being threatening. Here, a generally enhanced responsiveness to emotional stimuli (Heim et al., 2000; Kendall-Tackett, 2000; Obradović et al., 2012; Voellmin et al., 2015) may be associated with facilitated attention toward individuals evaluated as being potentially threatening, which may help victims of childhood maltreatment prepare for a confrontation with their perpetrators.

To test this assumption, we applied a more ecologically valid test for the FICE in maltreated individuals by combining an evaluative conditioning task and a modified visual search task. In the evaluative conditioning task, we coupled still images of neutral faces with short videos of negative/disapproving evaluations vs. neutral statements of the same actors from the E. Vids video set (Blechert et al., 2013). The images of neutral faces were then used as stimuli in a FIC recognition task. Here, the negatively or neutrally associated familiar neutral faces were presented in a crowd of unfamiliar neutral faces. Both male and female faces were included in the crowd and no individuals were represented more than once in a display. We implemented a design that included the combination of negatively associated familiar neutral faces in an unfamiliar neutral crowd, neutrally associated familiar neutral faces in an unfamiliar neutral crowd, and trials where no familiar neutral face was present. Because all faces showed neutral emotional expressions, we asked the participants to determine whether they recognized faces from the task before or not. Response times (RTs) were used to detect whether neutral faces evaluated as being negative were recognized more quickly than neutral faces with a neutral evaluation.

The goal of the present study was to examine the differential contributions of various forms of childhood maltreatment to biases in facial emotion processing. In doing so, we aimed for a better understanding of mechanisms that may be linking different types of maltreatment to differential psychopathological outcomes (Danielson et al., 2005; Teicher et al., 2006; Lobbestael et al., 2010; Teicher and Samson, 2013). With respect to previous studies, we predicted that a facilitated attention toward neutral faces that were previously coupled with negative evaluations would be particularly associated with emotional forms of childhood maltreatment (Günther et al., 2015). This prediction is supported by the idea that the evaluations of neutral faces as threatening are particularly salient for individuals who were victims of subtler, emotional forms of victimization.

## Materials and Methods

### Participants

Participants were recruited through online advertisements and bulletins on the Bielefeld University campus advertising a study examining the consequences of personality traits on attention. Inclusion criteria were age between the ages of 18 and 65 and sufficient knowledge of German language (clearly able to understand the information and instructions). No further exclusion criteria were applied. The sample consisted of 67 participants (45 females, 67.2%). Almost all were right-handed (97%), ranging in age from 18 to 40 years with a mean of 24.01 (*SD* = 3.95). Each participant read and signed an informed consent form that was approved by the Ethics Committee of Bielefeld University (protocol number: EUB_2015-104). The demographic characteristics of the sample are presented in Table 1.

**TABLE 1:** Subject characteristics and mean values on the assessments (*N* = 67).

Age, *M* (*SD, range*)	24.01(3.95,18−40)
Gender, % *female* (*n*)	67.2 (45)
Family status, % *single* (*n*)	44.8 (30)
Educational level, % high school graduation and higher (*n*)	80.6 (54)
Symptoms of Depression^1^, *M* (*SD*)	9.76 (7.57)
General Psychopathology^2^, *M* (*SD*)	0.57 (0.44)
Trait Anxiety^3^, *M* (*SD*)	41.63 (11.13)
Childhood Trauma Questionnaire, *M* (*SD*)	36.66 (10.89)
Emotional Abuse, *M* (*SD*)	8.54 (3.53)
Emotional Neglect, *M* (*SD*)	9.54 (4.00)
Physical Abuse, *M* (*SD*)	5.90 (1.40)
Physical Neglect, *M* (*SD*)	7.15 (2.58)
Sexual Abuse, *M* (*SD*)	5.52 (2.27)

*^1^Beck Depression Inventory; ^2^Brief Symptom Inventory - Global Severity Index; ^3^State Trait Aniety Inventory-Trait.*

### Stimuli, Design, and Apparatus

For the conditioning of neutral faces to negative/disapproving vs. neutral valence, we utilized a social conditioning paradigm using 3000 ms duration videos of negative and neutral sentences from the E. Vids video set (Blechert et al., 2013). Within this paradigm, still images of neutral faces of 4 different actors (2 female) served as conditioned stimuli (CSs) predicting dynamic videos of negative/disapproving evaluations (e.g., ‘You’re ridiculous,’ ‘I hate you,’ ‘I can’t bear you’) vs. neutral statements (e.g., ‘The bus is stopping,’ ‘It’s windy outside,’ ‘It’s 4 o’clock’) of the same actors as unconditioned stimuli (USs) (for details see Wiggert et al., 2017). No information about CS-US contingencies was provided. The conditioning consisted of 64 trials, 32 trials (16 per actor) coupling CSs with a negative US and 32 trials (16 per actor) coupling CSs with a neutral US. Each of the four actors spoke 8 different sentences, each presented twice. Video volume was constant across participants. Each trial started with the presentation of a black fixation cross in the center of a white screen for 500 ms before being replaced by the CS. CSs were shown for 1000 ms and were followed by the presentation of a black fixation cross in the center of a white screen for 1500 ms. Then, the USs were presented for 3000 ms. Inter-trial intervals varied randomly between 5000 and 7000 ms. Stimuli were presented on a 23-inch LCD monitor with a resolution of 1920 × 1080 pixel and 120 Hz refresh rate, using E-Prime 2.0 (Psychology Software Tools, Inc., Sharpsburg, PA, United States).

In the FIC recognition task, stimuli were 18 still images of neutral faces taken from the E. Vids video set (Blechert et al., 2013). Each image was 5.5 cm (width) × 6 cm (height). For each trial, nine images were presented simultaneously in a 3 × 3 matrix measuring 20.5 cm (width) × 18 cm (height). Participants viewed stimuli at a distance of 60 cm. Stimuli were presented on a 23-inch LCD monitor with a resolution of 1920 × 1080 pixel and 120 Hz refresh rate. We used the software package Inquisit 4.0.3 (Millisecond Software, Seattle, WA, United States) to deliver stimuli and record responses and reaction times (RTs). Responses were made on an external keyboard in which two keys were activated.

The FIC recognition task consisted of two blocks of 72 trials each, with a short break between the blocks. One quarter (36) of the matrices were target trials composed of eight unfamiliar neutral faces and one negative associated familiar neutral face that was derived from the conditioning task presented before. One quarter (36) of the matrices were target trials that consisted of eight unfamiliar neutral faces and one neutral associated familiar neutral face that was derived from the conditioning task presented before. The remaining half of the matrices (72) were target-absent trials composed of 9 unfamiliar neutral faces. In each matrix, participants were instructed to indicate as quickly and as accurately as possible whether they recognized an individual from the task before. Matrices were presented in random order. Two images of the same individual were never included in a matrix. Image positions within each matrix were randomly assigned. Each trial started with the presentation of a black fixation cross in the center of a white screen for 500 ms before being replaced by the stimulus matrix. Stimuli matrices were shown continuously until the participants’ response with an intertrial interval between 500 and 1000 ms. No feedback on accuracy was provided.

### Instruments

Childhood maltreatment was assessed using the German Version of the standard and well validated Childhood Trauma Questionnaire (CTQ; Wingenfeld et al., 2010; Klinitzke et al., 2012). The CTQ allows the assessment of all common types of childhood maltreatment (emotional abuse, emotional neglect, physical abuse, physical neglect, and sexual abuse) that have happened before the age of 18. The strength of the CTQ is that it allows for a severity rating of subtypes of maltreatment reflecting the fact that the different forms of child maltreatment may be continuous phenomena ranging from small to severe transgressions rather than clearly delimitable entities (Bernstein et al., 2003). In addition, applying empirically derived and externally validated cut off criteria established by Walker et al. (1999) provides a more accurate and clinically significant evaluation of the presence of maltreatment (Iffland et al., 2013). However, in the present study, dimensional sumscores of each CTQ subscale were used in the statistical analyses, while cut off scores by Walker et al. (1999) were only used to present prevalence rates of maltreatment types in the current sample. Because it was highly correlated with the other CTQ subscales, and presented with a weak internal consistency in comparison to the other subscales in a validation study (Klinitzke et al., 2012), the CTQ subscale physical neglect was not included in the following statistical analyses. However, to allow for a comparison with other samples, mean score and frequency of the CTQ subscale physical neglect are presented. In addition, the assessment battery included a socio-demographic questionnaire as well as well-established questionnaires for symptoms of depression (German version of the Beck Depression Inventory II, BDI-II; Hautzinger et al., 2006; Kühner et al., 2007), general psychopathology and psychological distress (German version of the Brief Symptom Inventory, BSI; Derogatis and Melisaratos, 1983; Derogatis, 1993; Franke, 2000), and trait anxiety (German version of the State Trait Anxiety Inventory-Trait, STAI-T; Spielberger et al., 1970; Laux et al., 1981).

### Procedure

Prior to the laboratory session, participants were asked to complete the assessment battery. Afterward, participants were tested individually in a darkened room. Instructions for the tasks were presented on the computer screen for the participants to read. Participants were informed that they would see a series of images and videos of different people and they would be asked to evaluate them. To control for baseline differences in the evaluation of the four actors that were presented in the social conditioning paradigm, subjects evaluated neutral still images of the actors (for details see Wiggert et al., 2017) for valence, arousal, and disapproval using an on-screen visual analog scale during a pre-conditioning rating phase. Next, participants underwent the 64 trials of the social conditioning paradigm. Then, a post-conditioning evaluative rating phase of each actor’s still image was obtained using the same rating scales as described above. Post-conditioning evaluative rating served to evaluate if social conditioning was successful. Before the FIC recognition task, participants were informed that they would see matrices consisting of several faces of different people and that their task was to indicate whether they recognized people from the earlier presented videos in the matrices. Participants indicated their response by pressing either the button “E” or “I” on a keyboard with their index fingers of both hands. Assignment of buttons (correct detection of a familiar face vs. correct rejection) alternated between blocks. Then, participants underwent the 144 trials of the FIC recognition task. After the FIC recognition task, another rating phase was completed, with the same steps as the pre- and post-conditioning phase. This was used to examine if the effect of social conditioning lasted throughout the FIC recognition task. After these tasks were completed, participants were debriefed.

### Data Reduction and Statistical Analyses

For the planned multiple linear regression analyses using RTs as outcome variable, a statistical power analysis was performed for sample size estimation using G^∗^Power 3.1 (Faul et al., 2009). With respect to the results reported by Günther et al. (2015), the effect size (ES) in this study was considered to be large using Cohen (1988) criteria (Cohen’s *f*^ 2^ = 0.35). With an alpha = 0.05 and power = 0.95 and inclusion of 6 predictors (emotional abuse, emotional neglect, physical abuse, sexual abuse, age, gender), the projected sample size needed with this effect size was *N* = 67. We anticipated a loss of data due to error trials and outliers of approximately 10 percent. Therefore, we aimed at recruiting 74 participants.

For the applied FIC recognition task, absolute RTs for the three conditions, i.e., correct detection of a negative associated familiar face, correct detection of a neutral associated familiar face, and correct rejection, were used to identify relative attentional biases in association with the different types of childhood maltreatment. Moreover, consistent with prior research (Gilboa-Schechtman et al., 1999), a relative attentional bias score was calculated by subtracting RTs of the correct detection of negatively associated familiar neutral faces from the RTs of the correct detection of neutrally associated familiar neutral faces (RTs correct detection of neutral associated faces – RTs correct detection of negative associated faces). Positive scores indicated a faster correct detection of negatively associated familiar faces.

Consistent with procedures utilized in prior studies (Gilboa-Schechtman et al., 1999; Pinkham et al., 2010; De Voogd et al., 2014), only correct responses were included in the RT analyses. Out of 144 trials, participants showed between 0 and 51 wrong responses. Seven participants were excluded due to error rates greater than 25% (more than 36 error trials). Accordingly, the remaining sample for the analyses consisted of 67 participants. Of all trials, 9,6% were error trials. Moreover, RT outliers, defined as ±2 SDs from the individual’s mean (2.3% of all correct trials), were excluded.

All statistical analyses were carried out using the Statistical Package for the Social Sciences (SPSS) 25. To present prevalence rates of maltreatment types in our sample, we applied cut off scores established by Walker et al. (1999) for the CTQ subscales to decide whether the dichotomous criteria of different types of child abuse were fulfilled. Maltreatment was assumed when threshold scores for emotional abuse (10), emotional neglect (15), physical abuse (8), physical neglect (8), and sexual abuse (8) were met. Moreover, to evaluate severity of abuse and neglect, cut off scores established by Bernstein et al. (2003) were applied.

Consistent with procedures utilized in prior research (Iffland et al., 2018), we calculated an experiential rating composite score (mean-score of the arousal, valence, and disapproval ratings) for analyses of differential conditioning effects on self-report data. Experiential data was assessed through a 2 (CS-type: CS-negative, CS-neutral) × 3 (time of assessment: pre-conditioning, post-conditioning, post FIC recognition task) analysis of variance (ANOVAs) with repeated measures on CS-type and time of assessment. When necessary, additional *post hoc t*-tests were conducted separately for different times of assessment. When Mauchly’s test indicated violation of the sphericity assumption, Greenhouse–Geisser corrections were applied and the original degrees of freedom together with Greenhouse–Geisser ε are reported.

Next, we utilized multiple linear regression models to examine the specific association of different types of childhood maltreatment and the indices of the FIC recognition task. For this purpose, we used the continuous sumscores of the CTQ subscales emotional abuse, emotional neglect, physical abuse, and sexual abuse. Regression analyses were conducted separately for each of the five indeces of the FIC recognition task presented above. Preliminary analyses showed no violation of the assumption of multicollinearity (tolerances > 0.46; variance inflation factors < 2.18).

## Results

The average age of the total sample was *M* = 24.01 (*SD* = 3.95). Table 1 presents participants’ means on the assessments. Out of the sample of 67 healthy individuals, we found 35 participants (52.2%) meeting Walker’s threshold severity criteria for at least one type of childhood abuse or neglect, as measured by the CTQ subscales (Walker et al., 1999). Threshold levels were met for emotional abuse for 25.4% (*n* = 17), emotional neglect for 13.4% (*n* = 9), physical abuse for 13.4% (*n* = 9), physical neglect for 34.3% (*n* = 23) and sexual abuse for 6.0% (*n* = 4). There were 32.8% (*n* = 22) meeting threshold levels for one subtype, 6.0% (*n* = 4) met threshold levels for two subtypes, 7.5% (*n* = 5) for three subtypes, 4.5% (*n* = 3) for four subtypes, and 1.5% (*n* = 1) for all five subtypes. In addition, Table 2 presents frequencies of abuse and neglect when applying severity cut-off scores established by Bernstein et al. (2003).

**TABLE 2:** Frequency of abuse and neglect when applying severity cut-off scores established by Bernstein et al. (2003).

**CTQ scale**	**Emotional abuse**	**Emotional neglect**	**Physical abuse**	**Physical neglect**	**Sexual abuse**
Severity					
None to minimal, % (*n*)	58.2(39)	58.2(39)	86.6(58)	65.7(44)	89.6(60)
Slight to moderate, % (*n*)	35.8(24)	28.4(19)	7.5(5)	16.4(11)	4.5(3)
Moderate to severe, % (*n*)	1.5(1)	10.4(7)	6.0(4)	11.9(8)	3.0(2)
Severe to extreme, % (*n*)	4.5(3)	3.0(2)	−(0)	6.0(4)	3.0(2)

To test whether the association of the neutral faces with negative and neutral valences was successful, a CS-type × Time of assessment ANOVA using the experiential rating composite score was conducted. The ANOVA revealed significant main effects of CS-type and time of assessment [CS-type: *F* (1, 66) = 119.09; *p* < 0.001; *η^2^* = 0.643; time of assessment: *F* (2, 132) = 44.34; *p* < 0.001; *η^2^* = 0.402; ε = 0.84]. Moreover, a significant interaction of CS-type and time of assessment was found [*F* (2, 132) = 70.24; *p* < 0.001; *η^2^* = 0.516]. While there were no significant differences in experiential ratings of the neutral faces before the conditioning task [*t*(66) = 0.55, *p* = 0.581], *post hoc t*-Tests showed that experiental ratings of the negatively associated familiar faces were rated significantly more negative than the neutrally associated familiar faces immediately after the conditioning task as well as after the attention paradigm [post-conditioning: *t*(66) = 13.09, *p* < 0.001; post FIC recognition task: *t*(66) = 7.54, *p* < 0.001]. Means and standard deviations on self-report data are presented in Table 3.

**TABLE 3:** Means and standard deviations on experiential rating composite scores (*N* = 67).

	**Pre-conditioning**	**Post-conditioning**	**Post FIC recognition task**
Negatively associated neutral faces, *M* (*SD*)	41.84^*a*^ (12.78)	74.29^*a*^ (17.88)	62.34^*a*^ (18.52)
Neutrally associated neutral faces, *M* (*SD*)	40.71^*a*^ (12.24)	28.97^*b*^ (14.84)	34.83^*b*^ (16.70)

*Means in the same column sharing the same superscript letter do not differ significantly from one another at *p* ≤ 0.05.*

Mean reaction times and correct detection rates are presented in Table 4. Bivariate Pearson correlation coefficients of different types of maltreatment and the indices of the FIC recognition task are presented in Table 5. The impact of the subtypes of childhood maltreatment on the RTs in the FIC recognition task was examined in gender- and age-adjusted regression analyses (see Table 6). Emotional abuse and emotional neglect significantly predicted the RTs of recognizing a negatively associated familiar face in a crowd of unfamiliar neutral faces [Full model: *F* (6, 60) = 4.39; adjusted *R*^2^ = 0.236; *p* = 0.001]. Here, emotional abuse showed a negative association, while emotional neglect was positively related to the detection of negatively associated stimuli. Emotional neglect also significantly predicted the RTs of recognizing a neutrally associated familiar face in a crowd of unfamiliar neutral faces [Full model: *F* (6, 60) = 2.98; adjusted *R*^2^ = 0.153; *p* = 0.013]. Moreover, emotional abuse and physical abuse significantly predicted the contrast of RTs of correct detection of negatively associated vs. neutrally associated familiar faces in a crowd of unfamiliar neutral faces. However, only a tendency toward significance was found for this regression model [Full model: *F* (6, 60) = 1.91; adjusted *R*^2^ = 0.077; *p* = 0.094]. Finally, the regression model examining the RTs for correct rejection did not achieve significance [Full model: *F* (6, 60) = 1.08; adjusted *R*^2^ = 0.007; *p* = 0.387].

**TABLE 4:** Mean RTs in milliseconds and standard deviations of the FIC recognition task (*N* = 67).

	**Correct detection rate**	**Reaction time in ms**
	
	**%**	***M* (*SD*)**
Detection of negatively associated neutral faces	86.4	1866.51 (319.39)
Detection of neutrally associated neutral faces	82.2	1897.22 (294.20)
Rejection of familiar faces	93.7	2936.79 (533.50)

**TABLE 5:** Bivariate Pearson correlation coefficients of different types of maltreatment and the indices of the FIC recognition task (*N* = 67).

	**Negative**	**Neutral**	**Negative vs.**
	
	**stimuli**	**stimuli**	**neutral stimuli**
	***r***	***r***	***R***
Emotional abuse	–0.13	0.08	0.28*
Emotional neglect	0.25*	0.38**	0.14
Physical abuse	0.31*	0.23	–0.14
Sexual abuse	0.26*	0.28*	0.00

*p < 0.05, **p < 0.01, ***p < 0.001.

**TABLE 6:** The association of different types of maltreatment and the indices of the FIC recognition task (*N* = 67).

	**Negative stimuli**					**Neutral stimuli**					**Negative vs. neutral stimuli**				
			
	***r*_*p*_**	**β**	**β 95%CI**	***T***	***p***	***r*_*p*_**	**β**	**β 95%CI**	***T***	***p***	***r*_*p*_**	**β**	**β 95% CI**	***T***	***p***s
Emotional abuse	−0.41*	−0.50*	[−0.79, −0.21]	–3.44	0.001	–0.21	–0.26	[−0.56, 0.05]	–1.67	0.101	0.29*	0.37*	[0.05, 0.69]	2.30	0.025
Emotional neglect	0.28*	0.36*	[0.04, 0.67]	2.23	0.029	0.33*	0.46*	[0.12, 0.79]	2.74	0.008	0.07	0.09	[−0.26, 0.44]	0.54	0.591
Physical abuse	0.20	0.22	[−0.06, 0.49]	1.54	0.129	–0.02	–0.02	[−0.32, 0.27]	–0.15	0.878	−0.27*	−0.33*	[−0.63, −0.02]	–2.13	0.037
Sexual abuse	0.10	0.11	[−0.16, 0.38]	0.81	0.422	0.10	0.11	[−0.18, 0.39]	0.75	0.457	–0.01	–0.02	[−0.32, 0.28]	–0.11	0.917

All multiple linear regression models are adjusted for age and gender. In all models, age and gender did not predict the outcome significantly. *p < 0.05; rp – partial correlation coefficients controlling for the effect of the other types of maltreatment. Negative stimuli – RTs of correct detection of a negatively associated familiar face in a crowd of unfamiliar neutral faces; Neutral stimuli – RTs of correct detection of a neutrally associated familiar face in a crowd of unfamiliar neutral faces; Negative vs neutral stimuli – contrast of RTs of correct detection of a neutrally associated familiar face in a crowd of unfamiliar neutral faces and RTs of correct detection of a negatively associated familiar face in a crowd of unfamiliar neutral faces: higher scores indicate a faster recognition of negatively associated familiar faces.

## Discussion

Using a FIC recognition task, the current study sought to examine differential associations of various forms of childhood maltreatment with attentional biases in facial emotion processing. In a sample of healthy participants, emotional abuse was associated with faster recognition of negatively associated faces. Experiences of emotional neglect were related to slower detection of both negative and neutral familiar faces in an unfamiliar crowd. Sexual abuse did not have an impact on the performance in the recognition task. In an analysis showing only a trend toward significance, however, physical abuse predicted slower detection of negatively compared to neutrally associated faces.

Our findings were consistent with previous research indicating associations of childhood maltreatment with altered attentional processes and emotion processing (Field et al., 2001; Pollak and Sinha, 2002; Gibb et al., 2009; Fani et al., 2011; Grant et al., 2011; Dannlowski et al., 2013; van Harmelen et al., 2013; Günther et al., 2015; Iffland et al., 2019). In line with previous findings (Pollak et al., 2000; Günther et al., 2015) and supporting our assumptions, emotional forms of maltreatment, in particular, were shown to be associated with shifted sensitivity in detecting negatively associated faces. **However**, attentional processes varied as a function of different forms of childhood maltreatment, indicating facilitated as well as impeded recognition of emotional faces.

In particular, the hypothesized facilitated attention toward negatively associated neutral faces relative to neutrally associated faces was specifically related to experiences of emotional abuse. Accordingly, in a prior study maintained attention toward sad faces was found to be more related to emotional forms of maltreatment than to physical and sexual abuse (Günther et al., 2015). Similarly, faster detection of negative faces in emotionally abused individuals was consistent with previous findings of greater sensitivity in detecting threatening cues from emotionally ambiguous faces in maltreated healthy individuals (Gibb et al., 2009). Although Gibb et al. (2009) did not explicitly examine the differential effects of each type of maltreatment, the high proportion of emotional maltreated participants may have influenced the direction of the effect.

Experiences of emotional neglect were associated with slower detection of familiar faces in a crowd of unfamiliar faces. Most notably, recognition was impeded for faces with both negative and neutral associations. In line with the above mentioned assumptions, our results may indicate a generalized attentional avoidance of personally salient emotional faces. With respect to a previous study using the same set of social-evaluative and neutral videos that reported similar psychophysiological responses to both kinds of videos in peer victimized participants (Iffland et al., 2018), it may be assumed that the perception of emotional valence of neutrally associated familiar faces is more negative in victims of emotional neglect enhancing the personal salience of the faces. From a developmental perspective, it has been assumed that awareness of emotional cues is enhanced by emotional experiences, allowing more efficient processing of emotional information (Dunn et al., 1991a,b; Pollak et al., 2000). As a consequence, because their parents’ emotional expressiveness was limited, victims of emotional neglect may show difficulties in discriminating emotional expressions impeding effective recognition and appropriate response to social cues. These difficulties may even be more pronounced when a personal salient face shows an emotionally indistinct expression. Instead, maladaptive emotion regulation processes, e.g., attentional avoidance, may be generalized to all kinds of emotional and personally salient faces. Accordingly, emotional maltreatment, particularly peer victimization, which is also characterized by neglectful behavior, has been linked to a general emotion-avoidant attentional style (Iffland et al., 2019). Similarly, Günther et al. (2015) indicated initial evidence of attentional avoidance of both positive and sad facial emotions in individuals with a more severe history of emotional maltreatment. Moreover, neglected children, albeit those who have experienced physical neglect, showed impaired emotion recognition abilites in prior research (Pollak et al., 2000). With respect to experiences of sexual abuse, our findings were consistent with prior reports indicating that sexual abuse was not associated with attentional biases when controlling for other forms of childhood maltreatment (Günther et al., 2015).

In our study, physical abuse was associated with impeded recognition of negatively associated faces when compared to neutrally associated faces in our study. This may be indicative of an attentional avoidance of negative stimuli in physically abused individuals. Accordingly, physical abuse has previously been reported to be predictive of the attentional avoidance of threatening faces in a visual-probe task (Pine et al., 2005). However, further studies are needed to bring the assumption of attentional avoidance as a consequence of physical abuse in line with prior research reporting that physical abuse was related to response biases for angry faces (Pollak et al., 2000). On the basis of less sensory information, children who have experienced physical abuse identified facial displays of anger more accurately suggesting facilitated access to representations of anger (Pollak and Sinha, 2002). In combination, it may be speculated that physical abuse initially heightens the perceptual sensitivity for threatening faces enabling an avoidant processing of potential physical threat. However, because the regression model investigating the contrast of RTs in recognizing negatively associated versus neutrally associated familiar faces in a crowd of unfamiliar neutral faces only trended toward significance, the results regarding the association of physical abuse and impeded recognition of negatively associated faces have to be considered with caution.

However, it is difficult to interpret attentional bias as measured by the visual search task. That is, impeded recognition of faces may be due to enhanced attention toward threat as well as a general delayed or avoidant response to threatening stimuli. Furthermore, aspects of difficulty disengaging and facilitated attention (also referred to as vigilance or attentional orienting), and attentional avoidance are not specifically addressed in the task. Therefore, further studies using a wide range of paradigms (e.g., dot probe task; MacLeod et al., 1986; Koster et al., 2004) and methods (e.g., eye tracking in addition to reaction times) are needed to examine the specific effects of different forms of maltreatment on information processing. Moreover, the additional use of positive stimuli would be benefitial to address the assumption of a generalized responding to all kinds of emotion in individuals who have experienced emotional neglect.

In our study, we extended previous research indicating differential associations between various forms of maltreatment and emotion processing (Pollak et al., 2000; Günther et al., 2015). Our findings may be indicative of the activation of different cognitive schemas that initiate interindividual differences in cognitive biases. Recently, it has been proposed that such cognitive schemas can be activated or primed through the induction of sad mood or stress (Scher et al., 2005; Brockmeyer et al., 2012; Günther et al., 2015). Hence, it is likely that these schema can also be elicited through reminders of adverse experiences. In line with this, emotional and physical maltreatment were associated with differentiated negative biases in self-associations and negative inferential styles (Gibb and Abela, 2008; van Harmelen et al., 2010; Wells et al., 2014). Thus, it may be assumed that varying forms of childhood maltreatment initially generate differing inferential styles and information processing biases. Through recurrence of maltreatment, these biases may generalize automatically and rigidly to new, initially unrelated situations (Rose and Abramson, 1992; Crick and Dodge, 1994). Similarly, a modified social-information-processing model proposed the activation of so-called victim schemas initiating hypervigilance for threatening cues and an attentional bias to threatening compared to non-threatening cues in social interactions (Rosen et al., 2007). According to this model, a victim schema is defined as a cognitive structure comprising an individual’s expectations, cognitions, emotions, and behavior that develop out of repeated patterns of interaction (Rosen et al., 2007). Thus, due to frequent activation, the processing of social information that was adaptively applied and learned in abusive environments because it may have helped to cope with negative emotions and negative outcomes for the individual’s well-being becomes more accessible. As a consequence, ambiguous situations (i.e., encountering neutral faces) will be more likely to be interpreted as being congruent with threatening situations because they activate the more readily accessible victim schema (Rosen et al., 2007). In line with this assumption, children who reported more frequent experiences of victimization responded more quickly to victim-related words in an Emotional Stroop task (Rosen et al., 2007). However, when encountering different types of maltreatment, different attentional, emotional, and behavioral processes may have initially been adaptive. Hence, there might be different victim schemas for all kinds of childhood maltreatment, reflecting specific information processing and emotion regulation pathways when confronted with abusive or neglecting environments. While facilitated recognition of socially threatening individuals may have enabled victims of emotional abuse to escape from potentially abusive situations, it may be speculated that attentional avoidance is an attempt to regulate negative emotions because no active coping or behavioral resources based on fight or flight stress responses were initially available in situations of emotional neglect (Williams et al., 1988; Mogg and Bradley, 1998; Bar-Haim et al., 2007; Iffland et al., 2019). This is in line with recent studies linking attentional avoidance to emotional regulation strategies (Mogg et al., 2004; Koster et al., 2005, 2006; Pflugshaupt et al., 2005; Cisler and Koster, 2010). To sum up, our results suggest that recurrent experiences of maltreatment determine the kind of attentional and emotional processing utilized when confronted with emotional faces. However, further research is needed to examine which mechanisms underlie the activation of interindividual victim schemas. Particularly, studies providing a detailed temporal resolution of information processing may be critical in reconciling the conflicting findings in the literature.

From a clinical perspective, repeated activation of victim schemas initializing attentional processes and emotion regulation may in turn inhibit accurate and adequate information-processing when no actual threat is present (Crick and Dodge, 1994; Rosen et al., 2007). As a consequence, the facilitated and easy accessibility of victim schemas may prevent individuals from adequately responding to social interactions, which could be one mechanism linking childhood maltreatment to the development of psychopathology documented in several studies (Danielson et al., 2005; Teicher et al., 2006; Lobbestael et al., 2010; Teicher and Samson, 2013).

Contrasting with previous studies applying the FIC paradigm (e.g., Pinkham et al., 2010), a modified visual search task was utilized in the present study. Instead of asking participants to indicate if, based on their facial expressions, all presented stimuli were from the same emotional category or if one was different, participants had to determine whether they recognized faces from the task before or not. Though, comparing our results to previous findings has to be done with caution because it remains unclear whether both kind of tasks refer to the same mechanisms. However, our study provides evidence that differential processing of threatening information does not rely on actual threatening expressions, but mental representations of threat through associations are sufficient to modify attentional processes. Although it remains an open question as to whether the negatively associated neutral faces in fact refer to earlier experiences of maltreatment, experiental ratings indicated that these were perceived as significantly more negative than the neutrally associated faces. This significantly more negative perception increased the likelihood of an activation of the proposed victim schemas or mental representations of maltreatment. Additionally, it may be assumed that emotion processing in survivors of childhood maltreatment is guided by mental representations than only by facial expressions, such as through the activation of victim schemas including anticipations of threat. It is likely that encountering a perpetrator or individuals resembling former perpetrators in appearance already triggers a cascade of attentional, emotional, and behavioral processes without the counterpart even having expressed, said, or done anything. Hence, the present study presents an ecologically valid attempt at better developing a clearer understanding of information processing in non-clinical individuals with varying degrees of childhood maltreatment.

In the present study, however, the set of stimuli may have contributed to the relatively stronger associations of emotional forms of maltreatment with changes in the processing of emotional information. It may be speculated that the present stimuli provide a social evaluative connotation that may be more strongly related to emotional forms of child maltreatment than to other forms of maltreatment. Hence, the smaller or null effects found for the influence of physical and sexual maltreatment on attentional biases may be due to the stimuli set utilized in the present study. Additionally, the stimulus set may not have been arousing or threatening enough to generate general attentional biases to the negatively associated neutral faces. Accordingly, no enhanced or impeded processing of negatively associated faces relative to neutrally associated faces was found in the total sample. Hence, the utilized combination of an evaluative conditioning task and a modified visual search task was not able to elicit a more efficient processing of negatively associated faces (Pinkham et al., 2010). However, it may speculated that less arousing and rather ambiguous stimuli are better suited to evoke differentiated processing in maltreated samples (Pollak and Sinha, 2002; Gibb et al., 2009), while clearly and strongly threatening stimuli could mask different processing. In this conceptualization, clearly and strongly threatening stimuli would lead to threat-related information-processing biases in all participants, irrespective of their history of childhood maltreatment. In maltreated children, however, even less arousing and rather ambiguous stimuli would lead to experience-specific information-pocessing biases because of an adaptively increased sensitivity to signals of danger (Pollak, 2003; Gibb et al., 2009).

Moreover, the present study has several additional limitations. The cross-sectional design and the assessment of childhood maltreatment based on retrospective accounts and self-report prohibit drawing conclusions about the causal relationship between maltreatment and altered emotion processing. In addition, retrospective assessment of adverse child experiences may be subject to recall biases (Häuser et al., 2011). However, it has been indicated that distortions of validity in retrospective assessments are not sufficiently large enough to invalidate retrospective studies (Hardt and Rutter, 2004). In addition, examining the aftermath of the full range of childhood maltreatment in adulthood often lacks valid alternatives to restrospective reports. For instance, contrasting with severe cases of physical and sexual abuse, particularly emotional forms of maltreatment are not reliably documented in child protection service, clinical, or medical records. To clarify causality in the association of childhood maltreatment and attentional biases, further research using longitudinal prospective designs is desirable. However, consistency in findings across different populations [i.e., children (Pollak et al., 2000), young adults (Gibb et al., 2009), and adults (Günther et al., 2015)] using different assessments of childhood maltreatment emphasizes on the robustness of associations of childhood maltreatment and biases in information-processing. Additionally, the generalizability of our findings is limited. The sample was relatively young and highly educated, with participants who were predominantly female. This should be addressed in future studies using larger and more representative samples. Further, the investigation of outcomes of childhood maltreatment on information processing in a sample of psychologically healthy individuals has both strengths and limitations. Given the strong association of childhood maltreatment and psychopathology (Danielson et al., 2005; Teicher et al., 2006; Lobbestael et al., 2010; Teicher and Samson, 2013), the results may not generalize to individuals with current or past psychopathology who are likely to have been exposed to a wide range of adversities. Particularly, information-processing may differ in victims of childhood maltreated with psychopathology and resilient victims of maltreatment. Even more, differences in information-processing and corresponding physiological reactivity may contribute to the development of psychopathology or resilience in victims of maltreatment (McLaughlin and Lambert, 2017). For instance, increased prefrontal regulation of amygdala reactivity was suggested to be a protective factor for the development of internalizing psychopathology following exposure to child adversities (Herringa et al., 2016). In line, differing effects of oxytocin on amygdala reactivity toward emotional faces were reported in trauma-exposed individuals with and without psychopathology suggesting a compensation of maladaptive information-processing in resilient individuals (Koch et al., 2016). However, in line with the findings of Wells et al. (2014), our study highlights the detrimental effects of maltreatment on attentional processes even in the absence of overt psychopathology.

While rates and mean scores of emotional neglect, physical abuse, physical neglect, and sexual abuse were comparable to frequencies, severity and mean scores of child maltreatment in a representative sample of the German population (Häuser et al., 2011; Iffland et al., 2013), results may have been influenced by a relatively large number of individuals with high levels of emotional abuse. Moreover, variance of the CTQ subscales differed within our sample (see table 1). Thus, the lack of significant associations for some kinds of maltreatment might be explained by restricted variability, particularly within the physical abuse scores. Therefore, replication of the current results in a sample with representative levels of childhood maltreatment exposure is needed. In addition, our study is limited by its focus on the unique effects of each type of maltreatment indicating differential associations between various types and emotion processing. However, different forms of maltreatment are inter-correlated and often co-occur (Häuser et al., 2011). In our sample, 37% of participants meeting criteria for one type of maltreatment also met criteria for at least one other type. Hence, it is likely that interactions between maltreatment types have an impact on emotion processing beyond the unique effects of each type. After having established the unique associations, therefore, future studies should aim at examining cumulative and interactive effects of types of maltreatment in larger representative samples. Moreover, exposure to adversities following childhood was not measured in the present study. With respect to studies reporting that childhood maltreatment increases the risk for experiencing adversities later in life (e.g., Dong et al., 2004; Finkelhor et al., 2007), it is likely that these later experiences are also associated with interindividual differences in attention processing. Hence, future studies should control for potential effects of additional adversities.

## Conclusion

Recently, it has been shown that childhood maltreatment retains cognitive scars even in healthy individuals (Wells et al., 2014). By altering processing of incoming emotional information, these scars may contribute to inadequate and maladaptive responding to social interactions setting individuals at risk for further victimization and latter psychopathology (Rosen et al., 2007; Masten et al., 2008; Fani et al., 2011; Wells et al., 2014). The results of the present study support previous research suggesting that childhood maltreatment, particularly emotional maltreatment, is associated with changes in attentional processes and emotion processing (e.g., Günther et al., 2015). More specifically, our findings indicate that the different kinds of childhood maltreatment are associated with differentiated cognitive and attentional outcomes. Hence, the different forms of maltreatment leave varying scars that may contribute to a wide range of inadequate reactions to social challenges. This may help to explain the broad range of psychopathology that is associated with maltreatment. Hence, a better understanding of the specific characteristics in emotion processing styles in the wake of childhood adversity could help to address short and long term consequences for victims. Therefore, the implementation of measures of childhood maltreatment in future studies on attentional processes in clinical as well as healthy samples is strongly suggested.

## Data Availability Statement

The datasets generated for this study are available on request to the corresponding author.

## Ethics Statement

The study involving human participants was reviewed and approved by the Ethics Committee of Bielefeld University. The participants provided their written informed consent to participate in this study.

## Author Contributions

BI participated in the conception and design of the study, collected data, performed the statistical analyses and interpretation of findings, and drafted the manuscript. FN participated in the conception and design of the study, made substantial contributions to the statistical analyses and interpretation of findings, helped to draft and revised the manuscript. All authors read and approved the final manuscript.

## Conflict of Interest

The authors declare that the research was conducted in the absence of any commercial or financial relationships that could be construed as a potential conflict of interest.
